# Supplementary material for: Overlapping caregiving demands and their association with poor subjective health and wellbeing and food insecurity among older rural South Africans
Source: PLOS Glob Public Health. 2025 Dec 8;5(12):e0004743. doi: 10.1371/journal.pgph.0004743 (PMC12685166; doi:10.1371/journal.pgph.0004743)
Supplement: S1 Table — (DOCX) [file pgph.0004743.s001.docx]

**S1 Table: Sociodemographic and wellbeing characteristics of caregivers and non-caregivers in 2018**

| **Caregiving category** | | | | | |
| --- | --- | --- | --- | --- | --- |
| **Characteristic** | **None** | **Adults only** | **Grandchildren and adults** | **Grandchildren**, | **p-value**^1^ |
| N | 2919 | 105 | 51 | 1101 |  |
| ***Covariates*** |  |  |  |  |  |
| Gender status, n (%) |  |  |  |  | <0.001 |
| Male | 1,480 (51%) | 37 (35%) | 14 (27%) | 331 (30%) |  |
| Female | 1,439 (49%) | 68 (65%) | 37 (73%) | 770 (70%) |  |
| Age category, n (%) |  |  |  |  | <0.001 |
| 40-50 | 479 (16%) | 27 (26%) | 11 (22%) | 84 (7.6%) |  |
| 51-60 | 732 (25%) | 39 (37%) | 17 (33%) | 275 (25%) |  |
| 61-70 | 737 (25%) | 22 (21%) | 13 (25%) | 378 (34%) |  |
| 71-80 | 567 (19%) | 13 (12%) | 8 (16%) | 259 (24%) |  |
| 81-120 | 404 (14%) | 4 (3.8%) | 2 (3.9%) | 105 (9.5%) |  |
| Marital status, n (%) |  |  |  |  | <0.001 |
| Never married | 292 (10%) | 14 (13%) | 1 (2.0%) | 33 (3.0%) |  |
| Currently married | 1,268 (43%) | 52 (50%) | 31 (61%) | 518 (47%) |  |
| Separated/Deserted/Divorced | 414 (14%) | 14 (13%) | 7 (14%) | 83 (7.5%) |  |
| Widowed | 945 (32%) | 25 (24%) | 12 (24%) | 467 (42%) |  |
| Household size, n (%) |  |  |  |  | <0.001 |
| Living alone | 463 (16%) | 6 (5.7%) | 0 (0%) | 20 (1.8%) |  |
| Living with one other person | 373 (13%) | 16 (15%) | 1 (2.0%) | 49 (4.5%) |  |
| Living in 3–6-person household | 1,255 (43%) | 53 (50%) | 19 (37%) | 488 (44%) |  |
| Living in 7+ person household | 828 (28%) | 30 (29%) | 31 (61%) | 544 (49%) |  |
| Education level, n (%) |  |  |  |  | <0.001 |
| No formal education | 1,339 (46%) | 26 (25%) | 19 (37%) | 481 (44%) |  |
| Some primary (1-7 years) | 944 (32%) | 43 (41%) | 17 (33%) | 435 (40%) |  |
| Some secondary (8-11 years) | 362 (12%) | 16 (15%) | 10 (20%) | 109 (9.9%) |  |
| Secondary or more (12+ years) | 274 (9.4%) | 20 (19%) | 5 (9.8%) | 76 (6.9%) |  |
| Employment status, n (%) |  |  |  |  | <0.001 |
| Not working | 2,395 (82%) | 76 (72%) | 40 (78%) | 933 (85%) |  |
| Homemaker | 45 (1.5%) | 5 (4.8%) | 0 (0%) | 13 (1.2%) |  |
| Employed (part or full time) | 479 (16%) | 24 (23%) | 11 (22%) | 155 (14%) |  |
| Wealth index class, n (%) |  |  |  |  | <0.001 |
| 1-Poorest | 609 (21%) | 20 (19%) | 9 (18%) | 206 (19%) |  |
| 2 | 605 (21%) | 18 (17%) | 8 (16%) | 190 (17%) |  |
| 3 | 596 (20%) | 15 (14%) | 8 (16%) | 203 (18%) |  |
| 4 | 570 (20%) | 18 (17%) | 13 (25%) | 229 (21%) |  |
| 5-Richest | 539 (18%) | 34 (32%) | 13 (25%) | 273 (25%) |  |
| ***Outcomes*** |  |  |  |  |  |
| Self-rated health, n (%) |  |  |  |  | <0.001 |
| Very good | 304 (10%) | 11 (10%) | 6 (12%) | 95 (8.6%) |  |
| Good | 1,366 (47%) | 59 (56%) | 27 (53%) | 589 (53%) |  |
| Moderate | 665 (23%) | 23 (22%) | 10 (20%) | 267 (24%) |  |
| Bad | 489 (17%) | 12 (11%) | 8 (16%) | 136 (12%) |  |
| Very bad | 80 (2.7%) | 0 (0%) | 0 (0%) | 14 (1.3%) |  |
| Unknown | 15 (0.5%) | 0 (0%) | 0 (0%) | 0 (0%) |  |
| Life dissatisfaction, Mean (SD) | 4.67 (1.99) | 4.30 (1.81) | 4.49 (1.83) | 4.72 (1.86) | 0.2 |
| Food security Mean (SD) | 0.64 (1.13) | 0.70 (1.21) | 0.44 (0.76) | 0.52 (0.95) | 0.13 |
| ^1^Pearson's Chi-squared test; Kruskal-Wallis rank sum test | | | | | |
