# Supplementary material for: Overlapping caregiving demands and their association with poor subjective health and wellbeing and food insecurity among older rural South Africans
Source: PLOS Glob Public Health. 2025 Dec 8;5(12):e0004743. doi: 10.1371/journal.pgph.0004743 (PMC12685166; doi:10.1371/journal.pgph.0004743)
Supplement: S2 Table — (DOCX) [file pgph.0004743.s002.docx]

**S2 Table: Ordered logistic regression models testing associations between demographic characteristics and health, food security, and life satisfaction in HAALSI sample wave 1**

|  | **Health status** | | **Food security** | | **Subjective Wellbeing** | |
| --- | --- | --- | --- | --- | --- | --- |
| **Characteristic** | **OR**^1^ | **95% CI**^1^ | **OR**^1^ | **95% CI**^1^ | **OR**^1^ | **95% CI**^1^ |
| Gender |  |  |  |  |  |  |
| Male | — | — | — | — | — | — |
| Female | 1.05 | 0.93, 1.19 | 0.81 | 0.69, 0.96 | 1.03 | 0.92, 1.16 |
| Age category |  |  |  |  |  |  |
| 40-50 | — | — | — | — | — | — |
| 51-60 | 1.29 | 1.10, 1.52 | 1.07 | 0.86, 1.33 | 1.10 | 0.94, 1.28 |
| 61-70 | 1.37 | 1.14, 1.64 | 0.62 | 0.49, 0.80 | 0.94 | 0.79, 1.11 |
| 71-80 | 2.00 | 1.63, 2.45 | 0.61 | 0.46, 0.81 | 1.13 | 0.93, 1.36 |
| 81-120 | 3.41 | 2.68, 4.34 | 0.56 | 0.40, 0.78 | 1.44 | 1.15, 1.81 |
| Marital status |  |  |  |  |  |  |
| Never married | — | — | — | — | — | — |
| Currently married | 0.86 | 0.67, 1.12 | 1.15 | 0.82, 1.63 | 0.99 | 0.78, 1.26 |
| Separated/Deserted/Divorced | 1.09 | 0.82, 1.44 | 1.26 | 0.88, 1.83 | 1.24 | 0.95, 1.61 |
| Widowed | 1.09 | 0.83, 1.43 | 1.36 | 0.96, 1.97 | 1.15 | 0.90, 1.49 |
| Household size |  |  |  |  |  |  |
| Living alone | — | — | — | — | — | — |
| Living with one other person | 1.07 | 0.84, 1.35 | 0.93 | 0.67, 1.29 | 0.90 | 0.72, 1.12 |
| Living in 3-6 person household | 0.83 | 0.68, 1.01 | 0.90 | 0.69, 1.17 | 0.92 | 0.76, 1.11 |
| Living in 7+ person household | 0.90 | 0.73, 1.11 | 0.85 | 0.64, 1.13 | 0.98 | 0.81, 1.20 |
| Education level |  |  |  |  |  |  |
| No formal education | — | — | — | — | — | — |
| Some primary (1-7 years) | 0.93 | 0.82, 1.06 | 1.07 | 0.90, 1.27 | 0.74 | 0.66, 0.84 |
| Some secondary (8-11 years) | 0.77 | 0.63, 0.93 | 1.04 | 0.80, 1.36 | 0.65 | 0.54, 0.78 |
| Secondary or more (12+ years) | 0.60 | 0.47, 0.75 | 0.91 | 0.64, 1.28 | 0.43 | 0.35, 0.53 |
| Employment status |  |  |  |  |  |  |
| Not working | — | — | — | — | — | — |
| Homemaker | 0.34 | 0.29, 0.41 | 0.51 | 0.39, 0.67 | 0.36 | 0.31, 0.43 |
| Employed (part or full time) | 0.55 | 0.47, 0.65 | 0.62 | 0.50, 0.78 | 0.91 | 0.78, 1.05 |
| Caregiving category |  |  |  |  |  |  |
| No caregiving duties | — | — | — | — | — | — |
| Caregiving for adults | 1.39 | 0.90, 2.12 | 1.21 | 0.66, 2.10 | 1.43 | 0.95, 2.14 |
| Caregiving for both grandchildren and adults | 1.41 | 0.88, 2.26 | 1.81 | 1.00, 3.15 | 1.33 | 0.86, 2.07 |
| Caregiving for grandchildren | 1.43 | 1.25, 1.63 | 2.22 | 1.86, 2.64 | 1.18 | 1.04, 1.33 |
| Wealth index class |  |  |  |  |  |  |
| 1 | — | — | — | — | — | — |
| 2 | 0.95 | 0.80, 1.12 | 0.68 | 0.56, 0.84 | 0.78 | 0.67, 0.92 |
| 3 | 1.06 | 0.89, 1.26 | 0.45 | 0.36, 0.56 | 0.70 | 0.60, 0.82 |
| 4 | 0.93 | 0.78, 1.10 | 0.34 | 0.27, 0.43 | 0.62 | 0.53, 0.73 |
| 5 | 0.82 | 0.68, 0.98 | 0.22 | 0.17, 0.29 | 0.61 | 0.52, 0.73 |
| No. Obs. | 4,856 |  | 4,721 |  | 4,856 |  |
| ^1^OR = Odds Ratio, CI = Confidence Interval | | | | | | |
