# Supplementary material for: Overlapping caregiving demands and their association with poor subjective health and wellbeing and food insecurity among older rural South Africans
Source: PLOS Glob Public Health. 2025 Dec 8;5(12):e0004743. doi: 10.1371/journal.pgph.0004743 (PMC12685166; doi:10.1371/journal.pgph.0004743)
Supplement: S3 Table — (DOCX) [file pgph.0004743.s003.docx]

**S3 Table. Ordered logistic regression models testing associations between demographic characteristics and health, food security, and life satisfaction in HAALSI sample wave 2**

|  | | **Health status** | |  | **Food security** | |  | **Subjective Wellbeing** | |
| --- | --- | --- | --- | --- | --- | --- | --- | --- | --- |
| **Characteristic** | | **OR^1^** | **95% CI^1^** |  | **OR^1^** | **95% CI^1^** |  | **OR^1^** | **95% CI^1^** |
| Gender status | |  |  |  |  |  |  |  |  |
| Male | | — | — |  | — | — |  | — | — |
| Female | | 1.09 | 0.96, 1.25 |  | 1.05 | 0.90, 1.24 |  | 1.07 | 0.95, 1.22 |
| Age category | |  |  |  |  |  |  |  |  |
| 40-50 | | — | — |  | — | — |  | — | — |
| 51-60 | | 1.08 | 0.88, 1.32 |  | 0.98 | 0.77, 1.23 |  | 1.43 | 1.19, 1.73 |
| 61-70 | | 1.48 | 1.20, 1.84 |  | 0.73 | 0.57, 0.94 |  | 1.67 | 1.36, 2.05 |
| 71-80 | | 2.72 | 2.15, 3.44 |  | 0.60 | 0.46, 0.80 |  | 2.73 | 2.18, 3.43 |
| 81-120 | | 4.40 | 3.38, 5.74 |  | 0.58 | 0.42, 0.79 |  | 3.86 | 2.96, 5.02 |
| Marital status | |  |  |  |  |  |  |  |  |
| Never married | | — | — |  | — | — |  | — | — |
| Currently married | | 0.95 | 0.75, 1.21 |  | 0.82 | 0.62, 1.07 |  | 0.56 | 0.44, 0.71 |
| Separated/Deserted/Divorced | | 0.93 | 0.71, 1.23 |  | 0.91 | 0.67, 1.24 |  | 0.73 | 0.56, 0.96 |
| Widowed | | 1.01 | 0.79, 1.30 |  | 0.82 | 0.62, 1.10 |  | 0.79 | 0.62, 1.01 |
| Household size | |  |  |  |  |  |  |  |  |
| Living alone | | — | — |  | — | — |  | — | — |
| Living with one other person | | 1.03 | 0.80, 1.32 |  | 0.99 | 0.74, 1.32 |  | 1.08 | 0.84, 1.37 |
| Living in 3-6 person household | | 0.93 | 0.76, 1.14 |  | 0.90 | 0.71, 1.15 |  | 1.21 | 0.99, 1.48 |
| Living in 7+ person household | | 0.88 | 0.71, 1.10 |  | 0.99 | 0.77, 1.28 |  | 1.11 | 0.90, 1.37 |
| Education level | |  |  |  |  |  |  |  |  |
| No formal education | | — | — |  | — | — |  | — | — |
| Some primary (1-7 years) | | 1.00 | 0.87, 1.14 |  | 0.73 | 0.62, 0.86 |  | 0.95 | 0.83, 1.08 |
| Some secondary (8-11 years) | | 1.07 | 0.87, 1.32 |  | 0.56 | 0.43, 0.72 |  | 0.92 | 0.76, 1.13 |
| Secondary or more (12+ years) | | 0.85 | 0.66, 1.10 |  | 0.41 | 0.29, 0.57 |  | 0.66 | 0.52, 0.83 |
| Employment status | |  |  |  |  |  |  |  |  |
| Not working | | — | — |  | — | — |  | — | — |
| Homemaker | | 0.69 | 0.42, 1.11 |  | 0.96 | 0.53, 1.65 |  | 0.54 | 0.35, 0.84 |
| Employed (part or full time) | | 0.58 | 0.48, 0.70 |  | 0.72 | 0.58, 0.90 |  | 0.75 | 0.63, 0.88 |
| Caregiving category | |  |  |  |  |  |  |  |  |
| No caregiving duties | | — | — |  | — | — |  | — | — |
| Caregiving for adults | | 0.92 | 0.64, 1.33 |  | 1.15 | 0.72, 1.79 |  | 0.91 | 0.64, 1.29 |
| Caregiving for both grandchildren  and adults | | 0.93 | 0.55, 1.57 |  | 0.86 | 0.45, 1.54 |  | 1.07 | 0.66, 1.73 |
| Caregiving for grandchildren | | 0.83 | 0.72, 0.95 |  | 0.90 | 0.75, 1.06 |  | 0.97 | 0.85, 1.10 |
| Wealth index class | |  |  |  |  |  |  |  |  |
| Poorest | | — | — |  | — | — |  | — | — |
| 2 | | 0.98 | 0.82, 1.18 |  | 0.77 | 0.63, 0.94 |  | 0.94 | 0.78, 1.12 |
| 3 | | 0.98 | 0.82, 1.18 |  | 0.58 | 0.47, 0.71 |  | 1.11 | 0.93, 1.32 |
| 4 | | 1.02 | 0.85, 1.23 |  | 0.44 | 0.35, 0.54 |  | 1.03 | 0.86, 1.23 |
| Richest | | 0.80 | 0.66, 0.97 |  | 0.35 | 0.27, 0.45 |  | 0.92 | 0.77, 1.11 |
| No. Obs. | | 4,161 |  |  | 3,775 |  |  | 3,908 |  |
|  |  | ^1^OR = Odds Ratio, CI = Confidence Interval | | | | | | | |
