# Supplementary material for: Overlapping caregiving demands and their association with poor subjective health and wellbeing and food insecurity among older rural South Africans
Source: PLOS Glob Public Health. 2025 Dec 8;5(12):e0004743. doi: 10.1371/journal.pgph.0004743 (PMC12685166; doi:10.1371/journal.pgph.0004743)
Supplement: Attachment — Submitted filename: Manuscript PGPH-D-25-01244.docx [file pgph.0004743.s004.docx]

**Dr Ibrahim A. Abdulganiyyu**
Department of Public and Environmental Health, Federal University Dutse,
[aaibrahim@fud.edu.ng](mailto:aaibrahim@fud.edu.ng)
12/6/2025

**To:**
Editorial Board
*PLOS Global Public Health*

**Subject:** Peer Review of Manuscript PGPH-D-25-01244 – *“Overlapping caregiving demands on older rural South Africans and their association with poor subjective health and wellbeing and food insecurity.”*

Dear Editors,

I have completed my review of the above manuscript and submitted my assessment for your consideration.

This manuscript addresses a timely and significant topic: the impact of caregiving responsibilities among older adults in a rural South African setting. It is a well-structured, methodologically sound study using high-quality longitudinal data (HAALSI), and it offers novel insights into the multidimensional burden experienced by informal caregivers.

The study makes a valuable contribution to the literature on aging, intergenerational caregiving, and public health in low-resource settings. It is particularly relevant in the context of policy discussions around social protection, gender equity, and support for caregivers in sub-Saharan Africa.

I recommend **minor revisions** to enhance the transparency of the statistical reporting, clarify methodological assumptions, and improve accessibility through a more straightforward presentation of effect sizes and visuals. These revisions are intended to strengthen an already commendable manuscript and improve its reach to both scholarly and policy audiences.

Please find my detailed comments and recommendation enclosed.

Sincerely,

**Ibrahim A. Abdulganiyyu PhD**

Department of Public and Environmental Health,

Faculty of Basic Medical Sciences

Federal University Dutse

## **Peer Review Summary and Recommendation**

### **Manuscript Title: “Overlapping Caregiving Demands on Older Rural South Africans and their association with poor subjective health and wellbeing and food insecurity”**

### **General Assessment:** This manuscript presents a timely and well-conceived study exploring the associations between overlapping caregiving responsibilities and three key outcomes — self-rated health, food insecurity, and subjective wellbeing — among older adults in rural South Africa. Using data from the robust HAALSI cohort, the authors present findings that are **both empirically rigorous and policy-relevant**.

The study addresses a notable gap in the caregiving literature by disaggregating caregiving roles and focusing on a **demographic that is systematically underrepresented in public health research**: older, rural caregivers in low- and middle-income countries. The analysis is methodologically sound, the writing is clear, and the implications are well-articulated. The work contributes meaningfully to the growing body of research on aging, intergenerational care, and social vulnerability in sub-Saharan Africa.

### **Strengths:**

- Strong conceptual framework grounded in relevant demographic, epidemiological, and social theory.
- Use of a high-quality, population-based **longitudinal dataset (HAALSI)**.
- Methodologically appropriate models (random effects ordered logistic regression).
- Clear public health relevance with well-expressed policy recommendations.
- References are current, relevant, and correctly cited.

### **Areas for Improvement (Minor Revision Suggested):**

1. **Results Reporting:**
   - Include more **numerical values (AORs and CIs)** in the narrative to better support key interpretations.
   - Consider adding one **visual representation** (e.g., a forest plot) to aid reader comprehension.
2. **Methods Transparency:**
   - Briefly mention whether the **proportional odds assumption** was tested in ordinal models.
   - Clarify how **missing data** were handled.
3. **Title and Abstract:**
   - Consider a slightly more concise title.
   - Add one or two **quantitative effect estimates** to the abstract to improve precision.
4. **Caregiving Measures:**
   - While acknowledged in limitations, it emphasize earlier that **intensity and duration** of caregiving were not measured.
5. **Reference Formatting:**
   - Add consistent **DOIs or persistent links** to all references where available.

### **Recommendation:** Minor Revision

This is a well-designed and vital study that warrants publication following minor revisions. The revisions suggested will improve clarity, strengthen methodological transparency, and enhance the paper’s accessibility to a broader audience of global health scholars and policymakers.
