# Supplementary material for: Overlapping caregiving demands and their association with poor subjective health and wellbeing and food insecurity among older rural South Africans
Source: PLOS Glob Public Health. 2025 Dec 8;5(12):e0004743. doi: 10.1371/journal.pgph.0004743 (PMC12685166; doi:10.1371/journal.pgph.0004743)
Supplement: Attachment — Submitted filename: Response to Reviewer Global Public Health - 21Jul2025.docx [file pgph.0004743.s006.docx]

**Title: Overlapping caregiving demands and their association with poor subjective health, wellbeing, and food insecurity among older rural South Africans**

Journal – PLOS Global Public Health PGPH-D-25-01244

We thank the reviewers and editor for their comments and the opportunity to revise and strengthen this manuscript. We have responded to all comments, as outlined below.

**Reviewer comments** are in bold text, our response is in normal text with *manuscript text in italics and* *additions underlined*.

**Reviewer 1**

**Reviewer comments**

**Reviewer #1: The paper is insightful and provides valuable additional information on the link between caregiving demands and the association to overall wellbeing in terms of health and food insecurity. The findings are insightful and are linked to the objectives of the research. However, there are a few things that need clarification or adjustments.**

We would like to thank Reviewer #1 for the thoughtful and constructive feedback on our manuscript. We are pleased that you found the paper insightful and valuable in contributing to the understanding of the link between caregiving demands and overall wellbeing, particularly in relation to health and food insecurity. Below we respond to the specific concerns you raised and describe the changes made in the revised manuscript to address them.

**1: Kindly indicate the justification and reason for using the gender-stratified random sample as well as the overall justification for the eligibility criteria for the study participants.**

Bearing in mind that we conducted a secondary data analysis, and thus were not directly involved in the original HAALSI sampling, we have provided our understanding of the original sampling intent. In many aging studies in sub-Saharan Africa, older men are underrepresented due to differential mortality, labor migration, or selective participation. By stratifying the sample by gender, the HAALSI study design ensured sufficient statistical power to detect gender-specific differences in key domains, including health outcomes and socioeconomic status. The age eligibility criteria aimed to include cohorts who were starting to face an increased risk of chronic health conditions, in a context where life expectancy in 2014 was around 60 years. We have added text to highlight these points in the Methods:

*“The inclusion of adults from age 40 onwards ensured that HAALSI captured health trajectories as individuals moved through middle-age towards increasing health risks. The gender-stratified random sampling approach was used to ensure adequate representation of both men and women across age groups in the study population, given that older men are often underrepresented in African aging studies due to differential mortality, labor migration, or selective participation.”*

**2.** **Indicate how you accounted for the unbalanced nature of the panel data set used in the study. What was the potential bias introduced and how and to what extent did it affect the final results. In addition in the model what was considered to be the fixed effects for the** study ?

To address this, the unbalanced nature of our panel data, we employed random-effects models at the individual level. These models are well-suited for unbalanced panel data, as they allow for intra-individual correlation over time and do not require equal observations across all individuals. This approach enabled us to include all available data points without biasing the sample toward only those with complete follow-up, thereby maximizing statistical power and improving generalizability. We note that non death loss to follow up between waves was only 5.7%.

We acknowledge that attrition may still introduce bias if the probability of dropout is systematically related to unobserved factors associated with our outcomes (e.g., individuals in poorer health being more likely to die or relocate). While we could not fully eliminate this source of potential selection bias, our model adjusts for a comprehensive set of observed covariates that are known predictors of attrition (e.g., age, gender, baseline health status), helping to mitigate its effects.

We have now included this explanation in the Methods section to clarify the modeling choices and their implications for our findings.

*“Our primary analysis included data from both waves with fixed effects for wave, age, gender, marital status, household size, education, employment status, caregiving status, and wealth index, and random effects for participants, to assess whether our results differed across study waves.”*

**Reviewer 2**

**Reviewer comments**

**Reviewer #2: Manuscript Title: “Overlapping Caregiving Demands on Older Rural South Africans and their association with poor subjective health and wellbeing and food insecurity”
General Assessment: This manuscript presents a timely and well-conceived study exploring the associations between overlapping caregiving responsibilities and three key outcomes — self-rated health, food insecurity, and subjective wellbeing — among older adults in rural South Africa. Using data from the robust HAALSI cohort, the authors present findings that are both empirically rigorous and policy-relevant.
The study addresses a notable gap in the caregiving literature by disaggregating caregiving roles and focusing on a demographic that is systematically underrepresented in public health research: older, rural caregivers in low- and middle-income countries. The analysis is methodologically sound, the writing is clear, and the implications are well-articulated. The work contributes meaningfully to the growing body of research on aging, intergenerational care, and social vulnerability in sub-Saharan Africa.
Strengths:
• Strong conceptual framework grounded in relevant demographic, epidemiological, and social theory.
• Use of a high-quality, population-based longitudinal dataset (HAALSI).
• Methodologically appropriate models (random effects ordered logistic regression).
• Clear public health relevance with well-expressed policy recommendations.
• References are current, relevant, and correctly cited.**

Thank you for this generous assessment. We are pleased that the purpose and contributions of our study were clearly conveyed and found to be relevant and timely. Your recognition of the strengths of our conceptual framework, data source, and modeling approach is greatly appreciated.

1. **Result reporting: include more numerical values (AORs and CIs) in the narrative to better support key interpretations.**

Thank you for this helpful suggestion. We have revised the Results sections to include more Cis, with a focus on emphasizing the findings that are most directly aligned with the study’s core objectives-namely, understanding the relationship between overlapping caregiving responsibilities and self-rated health, food insecurity, and subjective wellbeing among older adults. For example:

*“In our panel analysis (Table 2), caregiving responsibilities were significantly associated with greater food insecurity, statistically significant in the case of caregiving for grandchildren alone (OR 1.42; 95% CI: 1.26, 1.61). This pattern was similar for subjective wellbeing, with caregivers for grandchildren reporting lower life satisfaction. Poor self-reported health was positively associated with caregiving for grandchildren, as indicated by an OR of 1.09 (95% CI:1.01, 1.20), meaning these caregivers are more likely to report worse health.”*

**Results reporting: Consider adding one visual representation (e.g., a forest plot) to aid reader comprehension***.*

Thank you for this valuable suggestion. In response, we have added a forest plot to visually represent the associations between the covariates including the caregiving categories and the three key outcomes: self-rated health, food insecurity, and subjective wellbeing. This visual aid is intended to enhance reader comprehension by providing a clear summary of the adjusted odds ratios and 95% confidence intervals for each outcome across caregiving groups.

The forest plot is included as Figure 1 in the revised manuscript and is referenced in both the Results sections to help interpret and contextualize the findings.

2. **Methods Transparency: Briefly mention whether the proportional odds assumption was tested in ordinal models.**

Thank you for this important point. We tested the proportional odds assumption using the Brant test. While the test indicated some violations of the assumption in our models, we chose to retain the ordinal regression framework for several reasons.

First, ordinal models offer a parsimonious, interpretable, and theoretically coherent approach that aligns with the ordered nature of our outcomes. Second, there are limited modeling alternatives that allow for both random effects and partial proportional odds structures, particularly in the context of longitudinal data. Finally, research has shown that ordinal logistic regression is reasonably robust to minor violations of the proportional odds assumption and remains widely used in applied health and social science research (Agresti, 2010; Harrell, 2015; Fullerton, 2009).

We have added the following clarification to the Methods/ Analytical section of the manuscript:

*“The proportional odds assumption was assessed using the Brant test. Although the test indicated some violations, we retained the ordinal regression models due to their interpretability, coherence, and parsimony. Moreover, there are limited alternatives that accommodate both random effects and partial proportional odds structures. Despite these concerns, the proportional odds model is considered robust to minor violations and is commonly applied in similar research contexts (Liu et al., 2023).”*

**Methods transparency: Clarify how missing data were handled.**

Thank you for this important question. Missing data, particularly on the outcome variables, were handled using listwise deletion that is, observations with missing outcome data were excluded from the analysis. Given the relatively low proportion of missingness in key variables, this approach was deemed appropriate and unlikely to introduce substantial bias. We have clarified this approach in the Methods/ Analytical section of the revised manuscript.

*“Missing outcome data were handled by listwise deletion (i.e., observations with missing outcomes were excluded from the analysis).”*

3. **Title. Consider a slightly more concise title.**

Thank you for this helpful suggestion. We have revised the manuscript title to be more concise while retaining its clarity and key elements. The new title is:

*“Overlapping caregiving demands and their association with poor subjective health, wellbeing, and food insecurity among older rural South Africans.”*

We believe this title effectively captures the study focus in a clear and succinct manner.

**Abstract: Add one or two quantitative effect estimates to the abstract to improve precision.**

Thank you for this helpful suggestion. In response, we have revised the Abstract to include key quantitative effect estimates—specifically Odds Ratios (ORs) and 95% Confidence Intervals (CIs)—for the associations between caregiving for grandchildren and the three main outcomes: food insecurity, life satisfaction, and self-rated health.

*“Notably caregiving for grandchildren was associated with higher odds of food insecurity (OR: 1.42; CI:1.26, 1.61), lower life satisfaction* *(OR: 1.09; CI:1.01, 120), and reported worse health compared to non-carers (OR: 1.09; CI:1.01, 120).”*

4. **Caregiving Measures: While acknowledged in limitations, it emphasize earlier that intensity and duration of caregiving were not measured**.

While this measure captures the type of caregiving provided, we acknowledge that it does not include information on the intensity or duration of caregiving. We now emphasize this limitation earlier in the Methods section as well as in the Limitations section of the Discussion.

*“Participants were asked whether they had provided care in each of three domains in the past 12 months: (a) adult family members who are unable to carry out their basic daily activities; (b) grandchildren; and (c) parents or parents-in-law. From these responses we created a four-category variable: (i) caregiving for adults only; (ii) caregiving for grandchildren only;, (iii) caregiving for both adults and grandchildren and (iv) no caregiving). While this measure captures type of caregiving, it does not assess the intensity (e.g., hours per week) or duration (e.g., length of caregiving episode) of care provided. We acknowledge this as a limitation of the dataset and discuss its implications in the Limitations section.”*

*“Second, caregiving intensity and duration were not captured in detail, and these variables may influence the observed associations with health, food security, and well-being.”*

5. **Reference Formatting: Add consistent DOIs or persistent links to all references where available.**

All references were checked and all DOIs or similar added where available.
